# Astragaloside IV inhibits glucose-induced epithelial-mesenchymal transition of podocytes through autophagy enhancement via the SIRT–NF-κB p65 axis

**DOI:** 10.1038/s41598-018-36911-1

**Published:** 2019-01-23

**Authors:** Xiaolei Wang, Yanbin Gao, Nianxiu Tian, Tao Wang, Yimin Shi, Jiayi Xu, Bingjie Wu

**Affiliations:** 1 School of Traditional Chinese Medicine, Capital Medical University, 10 Youanmenwai, Xitoutiao, Fengtai District, Beijing, China; 2 Beijing Key Lab of TCM Collateral Disease theory Research, 10 Youanmenwai, Xitoutiao, Fengtai District, Beijing, China

## Abstract

Both autophagy and podocyte epithelial-mesenchymal transition (EMT) are critical factors in glomerular diseases that involve proteinuria and fibrosis. Here, we sought to determine whether plant-derived saponin astragaloside IV (AS-IV) was able to reverse renal fibrosis and improve renal function through regulation of autophagy and podocyte EMT. Cultured immortalized mouse podocytes and KK-Ay mice models of diabetes were exposed to AS-IV. Western blotting, real-time PCR, immunofluorescence and histochemistry were used to analyze markers of autophagy and podocyte EMT. We observed that AS-IV inhibited glucose-induced podocyte EMT and enhanced autophagy by decreasing NF-κB subunit p65 acetylation as well as increasing Sirtuin1 (SIRT1) expression. Treatment of the cells and animal models with a SIRT1 inhibitor EX527 was able to reverse these effects. The SIRT1 activator SRT1720 was also found to decrease p65 acetylation and enhance autophagy in glucose-induced podocyte EMT. Additionally, further treatment with autophagy inhibitor 3-methyladenine was able to reverse the effects of AS-IV on podocyte EMT, while the autophagy activator rapamycin or the NF-κB pathway inhibitor ammonium pyrrolidinedithiocarbamate (PDTC) were able to reverse glucose-induced podocyte EMT. Notably, both renal fibrosis and renal function in diabetic KK-Ay mice were improved after treatment with AS-IV. These findings support AS-IV as a renoprotective agent that likely exerts its effects on podocyte EMT through modulation of the SIRT1–NF-κB pathway and autophagy activation. Further studies are required to clarify the role of AS-IV as a potential therapeutic agent in glomerular diseases.

## Introduction

Diabetes is a disease that often targets end organ microvasculature, and is a significant cause of chronic kidney disease. Diabetic kidney disease (DKD) is histologically marked by the presence of renal fibrosis and the presence of clinical proteinuria^1^. Podocyte injury is a common feature accompanying fibrosis and proteinuria in many glomerular diseases, including DKD^2^. Epithelial-mesenchymal transition (EMT), a phenotypic transition of cells from the differentiated epithelial-like state to mesenchymal-like phenotype, is the underlying mechanism of podocyte injury in DKD^3–5^. In response to harmful stimuli, podocytes usually lose their differentiated morphology and epithelial markers like nephrin, podocin, and zonula occludens-1 (ZO-1), and acquire mesenchymal markers such as fibronectin (FN), fibroblast-specific protein-1 (FSP-1) and α-Smooth Muscle Actin (α-SMA)^3,6,7^. Excretion of these plasma proteins into the urine accelerates the progression of renal fibrosis in DKD.

Sirtuin 1 (SIRT1), a deacetylase that can regulate metabolism and cell survival^8,9^, is involved in the pathological processes that drive podocyte dysfunction^10^. Several transcription factors and proteins are regulated by SIRT1, including NF-κB. Overexpression of SIRT1 has been shown to augment NF-κB p65 subunit deacetylation and repress NF-κB transcription^11^. Exogenously administered SIRT1 reversed podocyte dysfunction in a podocyte-specific SIRT1 knockout diabetic mouse model^12^. However, the mechanism of how SIRT1 regulates podocyte EMT induced by high glucose concentrations is still not fully understood.

Autophagy, an evolutionarily conserved lysosomal pathway essential for cellular homeostasis which is involved in immunological diseases and cancer progression, is subjected to regulation by the NF-κB system. The NF-κB signaling pathway inhibits autophagy during high glucose induced podocyte apoptosis by downregulating LC3-II^13^. Previous studies have reported that autophagy may be directly regulated by SIRT1 in many cells, including podocytes. Huang *et al*. demonstrated that SIRT1 upregulated starvation-induced autophagy by promoting the deacetylation of nuclear LC3^14^. Several studies have reported a direct connection between autophagy and EMT in tumor cells^15–17^. Li *et al*. found that enhanced podocyte EMT is associated with dysfunctional lysosomes as characterized by a disrupted autophagic flux, which suggests that autophagy can also regulate EMT in podocytes^18^. However, the molecular mechanism of autophagy-associated podocyte EMT is still not clear.

**Figure 1:**
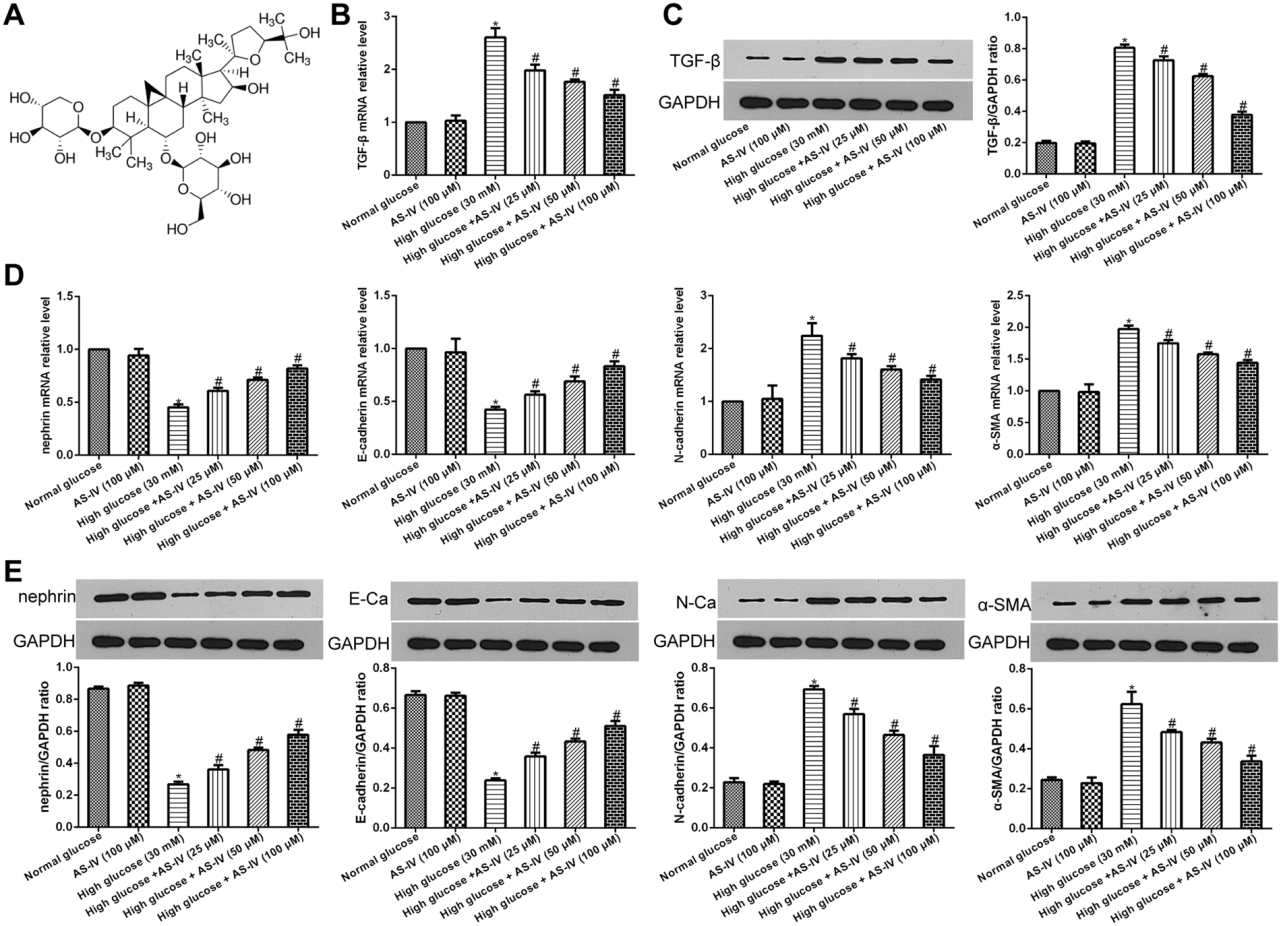
AS-IV effects on hyperglycaemia-triggered podocyte EMT. (**A**) AS-IV chemical structure. (**B–E**) Podocytes were pretreated with high/normal glucose for 1 hour, and then incubated with or without AS-IV (25, 50 and 100 μM) for 48 hours. (**B**) mRNA expression levels of TGF-β were detected using real-time PCR. (**C**) TGF-β protein levels were quantified using Western blotting. (**D**) mRNA expression levels of α-SMA, N-cadherin, E-cadherin and nephrin were detected using real-time PCR. (**E**) Protein levels of α-SMA, N-cadherin, E-cadherin and nephrin were detected using Western blotting. Note: E-Ca, E-cadherin; N-Ca, N-cadherin. The molecular weight of the proteins: TGF-β, 44 kDa; nephrin, 100 kDa; E-cadherin, 110 kDa; N-cadherin, 100 kDa; α-SMA, 42 kDa. Data is presented as mean ± SD. n = 3. *Compared with normal glucose cohort or AS-IV cohort, P < 0.05; ^#^compared with high glucose cohort, P < 0.05.

Accumulating evidence has demonstrated that Astragaloside IV (AS-IV) (C41H68O14, molecular weight = 784.97, Fig. 1A), a bioactive saponin extract of the *Astragalus* root, possesses a broad range of pharmacological effects, including anti-inflammatory and anti-tumor functions^19,20^. AS-IV has been shown to be able to alleviate podocyte oxidative stress and apoptosis by inhibiting ER Stress and enhancing autophagy in streptozotocin-induced diabetic mice^21^. Several studies have shown that podocyte EMT could be regulated by a variety of traditional Chinese medicine^22–24^. A previous research reported that AS-IV inhibited EMT by suppressing markers of oxidative stress in renal proximal tubular cells^25^. However, little is known regarding the impact of AS-IV on EMT in podocyte cells. Our investigation focuses on investigating the probable role of AS-IV in podocyte EMT, focusing specifically on the role of autophagy and SIRT1-facilitated NF-κB p65 subunit deacetylation.

## Results

### AS-IV effects on hyperglycemia-induced podocyte EMT

**Figure 2:**
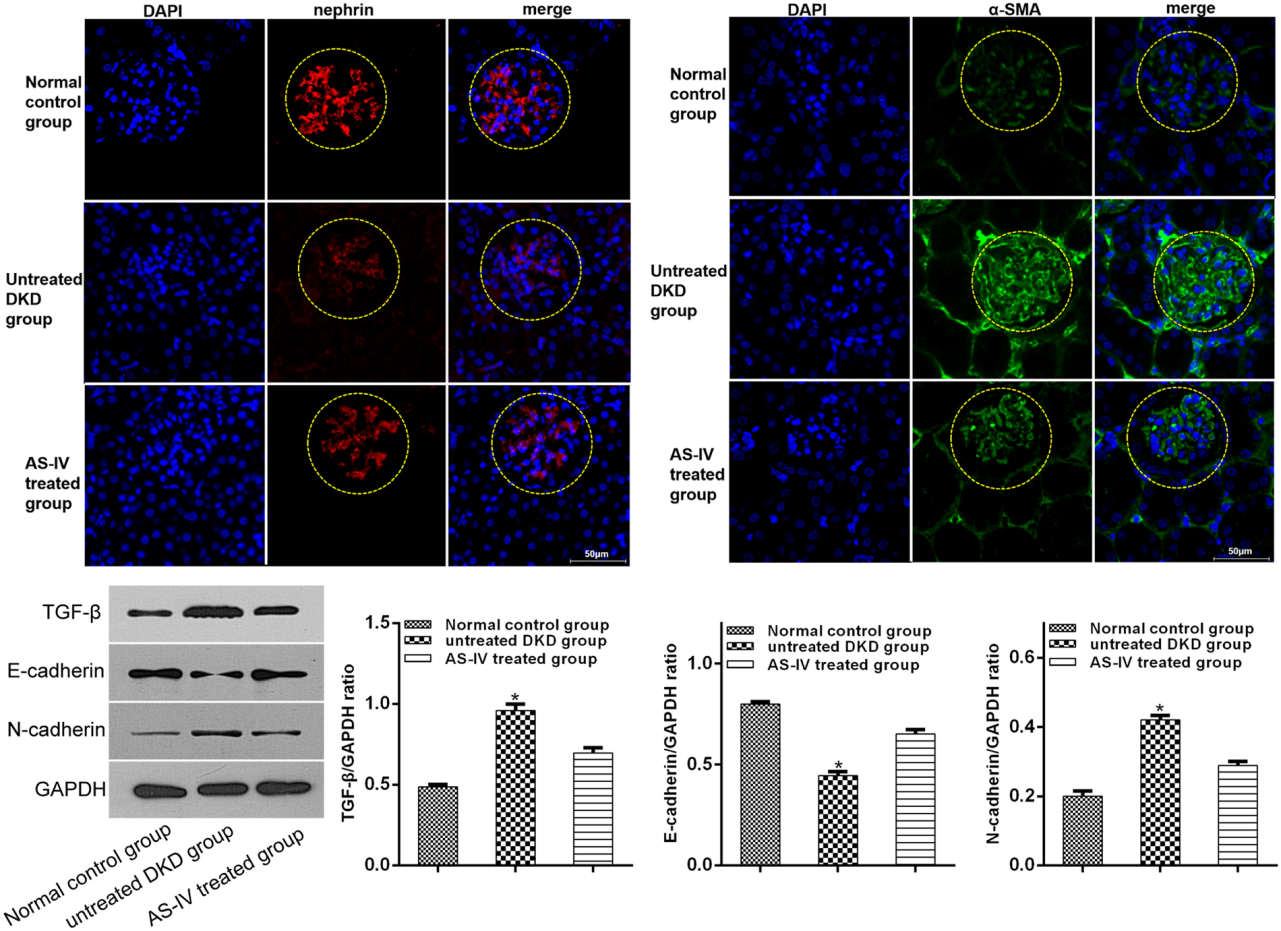
*In vivo* AS-IV effects on podocyte EMT. α-SMA and nephrin levels *in vivo* were analyzed by immunofluorescence assay and the levels of TGF-β, N-cadherin and E-cadherin were quantified using Western blotting. Data is presented as mean ± SD. n = 3. *Compared with normal control cohort, or AS-IV treated cohort, P < 0.05.

TGF-β is a critical factor in promoting fibrosis and is closely related to EMT^26^. Therefore, to further examine the impact of AS-IV on hyperglycemia-induced podocyte EMT, we first quantified TGF-β levels. AS-IV treatment (25, 50 and 100 μM) was found to significantly decrease TGF- β expression in podocytes exposed to glucose in a dose-dependent manner (Fig. 1B,C). Further analysis of key EMT markers, mainly E-cadherin, epithelial marker nephrin, α-SMA and the mesenchymal marker N-cadherin revealed that AS-IV significantly raised the levels of E-cadherin and nephrin while suppressing levels of α-SMA and N-cadherin in podocytes exposed to hyperglycemic conditions in a dose-dependent manner (Fig. 1D,E). The results suggested that high glucose concentrations promoted podocyte EMT which was subsequently reversed upon exposure to AS-IV in a dose-dependent manner. Furthermore, mice kidneys were also found to positively express markers of podocyte EMT. In conclusion, AS-IV treatment was able to suppress TGF- β, N-cadherin and α-SMA levels while simultaneously increasing levels of nephrin and E-cadherin in contrast to untreated DKD mice (Fig. 2).

### AS-IV effects on SIRT1 expression in hyperglycemia-induced podocyte EMT and functions of NF-kB

**Figure 3:**
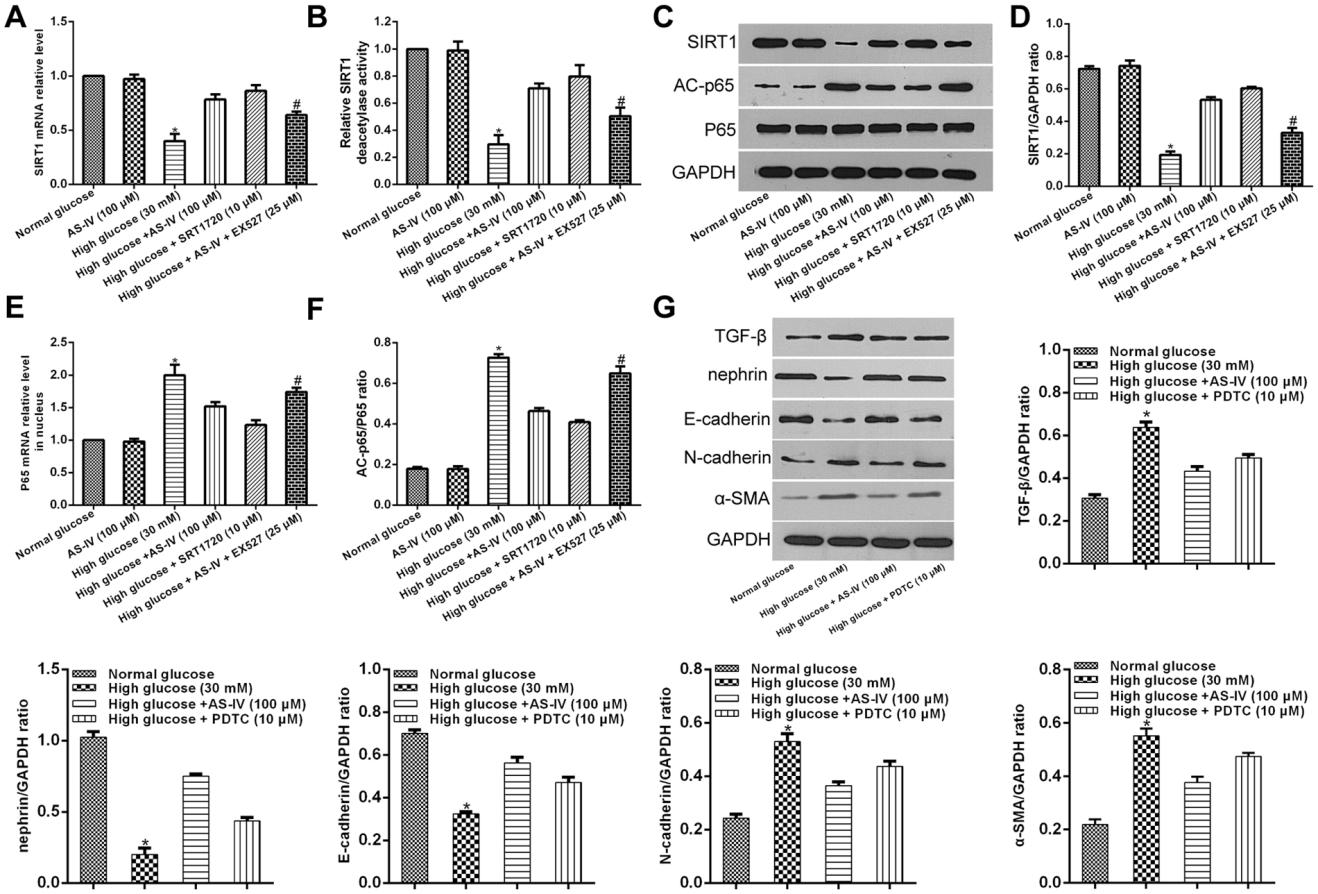
*In vitro* AS-IV effects on SIRT1 and NF-kB p65 expression in hyperglycaemia-triggered podocyte EMT. (**A–F**) Podocytes were exposed to either a SIRT1 inhibitor EX527 or SIRT1 activator SRT1720 after a 1 hour exposure to hyperglycaemic conditions, and were subsequently incubated with or without AS-IV (100 μM) for 48 hours. (**A**,**E**) mRNA expression levels of SIRT1and p65 were quantified using real-time PCR. (**B**) The deacetylase activity of SIRT1 was detected with a SIRT1 activity assay. (**C**,**D**,**F**) The protein levels of SIRT1 and AC-p65 were quantified using Western blotting. The molecular weight of the proteins: SIRT1, 110 kDa; AC-p65, 65 kDa; p65, 65 kDa. Data is presented as mean ± SD. n = 3. *Compared with normal glucose cohort, or AS-IV cohort, or high glucose plus AS-IV cohort, or high glucose plus SRT1720 cohort, P < 0.05; ^#^compared with high glucose plus AS-IV cohort, P < 0.05. (**G**) Podocytes were pretreated with high glucose for 1 hour, and then incubated with PDTC or AS-IV for 48 hours. The levels of TGF-β, nephrin, E-cadherin, N-cadherin and α-SMA were quantified with Western blotting. Data is presented as mean ± SD. n = 3. *Compared with normal glucose cohort, or high glucose plus AS-IV cohort, or high glucose plus PDTC cohort, P < 0.05.

To further explore the impact of AS-IV on SIRT1 expression in hyperglycemia-induced podocyte EMT, podocytes were first exposed to high or normal glucose concentrations for one hour followed by a 48 hour incubation period with or without AS-IV (100 μM). SIRT1 expressions were markedly low in hyperglycemia-exposed podocytes. This effect was reversed upon exposure to AS-IV treatment (Fig. 3A–D). In order to clarify how AS-IV affected SIRT1 expression, podocytes were first exposed high glucose conditions for one hour and subsequently exposed to either the SIRT1 activator SRT1720 or SIRT1 inhibitor EX527 and finally incubated with or without AS-IV (100 μM) for 48 hours. SRT1720 was found to markedly raise SIRT1 expression in hyperglycaemia-induced podocyte EMT and that EX527 abolished the effects of AS-IV. The changes of SIRT1 deacetylase activity were in accordance with the variations of SIRT1 expression. These findings confirmed that AS-IV reversed the decrease in SIRT1 in hyperglycaemia-triggered EMT in podocytes.

To clarify whether NF-κB p65 was involved in AS-IV-induced SIRT1 activity in hyperglycaemia-triggered podocyte EMT, the acetylated NF-κB p65 expressions were analyzed. High glucose increased p65 acetylation during podocytes EMT, which was reversed by AS-IV (100 μM) (Fig. 3C,E,F). The effect of AS-IV was reversed in podocytes treated with EX527. Furthermore, SRT1720 decreased p65 acetylation levels in podocytes that were cultured in a high glucose conditions. NF-κB effects on hyperglycaemia-triggered EMT of podocytes were investigated by subjecting podocytes to a 1 hour incubation period in high glucose conditions before treatment with AS-IV or ammonium pyrrolidinedithiocarbamate (PDTC, an inhibitor of NF-κB) for 48 hours. Data revealed that PDTC treatment attenuated TGF-β, N-cadherin and α-SMA levels while increasing expressions of podocyte nephrin and E-cadherin levels, findings that were consistent with AS-IV treatment (Fig. 3G). Taken together, AS-IV was found to mediate hyperglycaemia-triggered EMT in podocytes through SIRT1 deacetylation of NF-κB p65 subunit.

**Figure 4:**
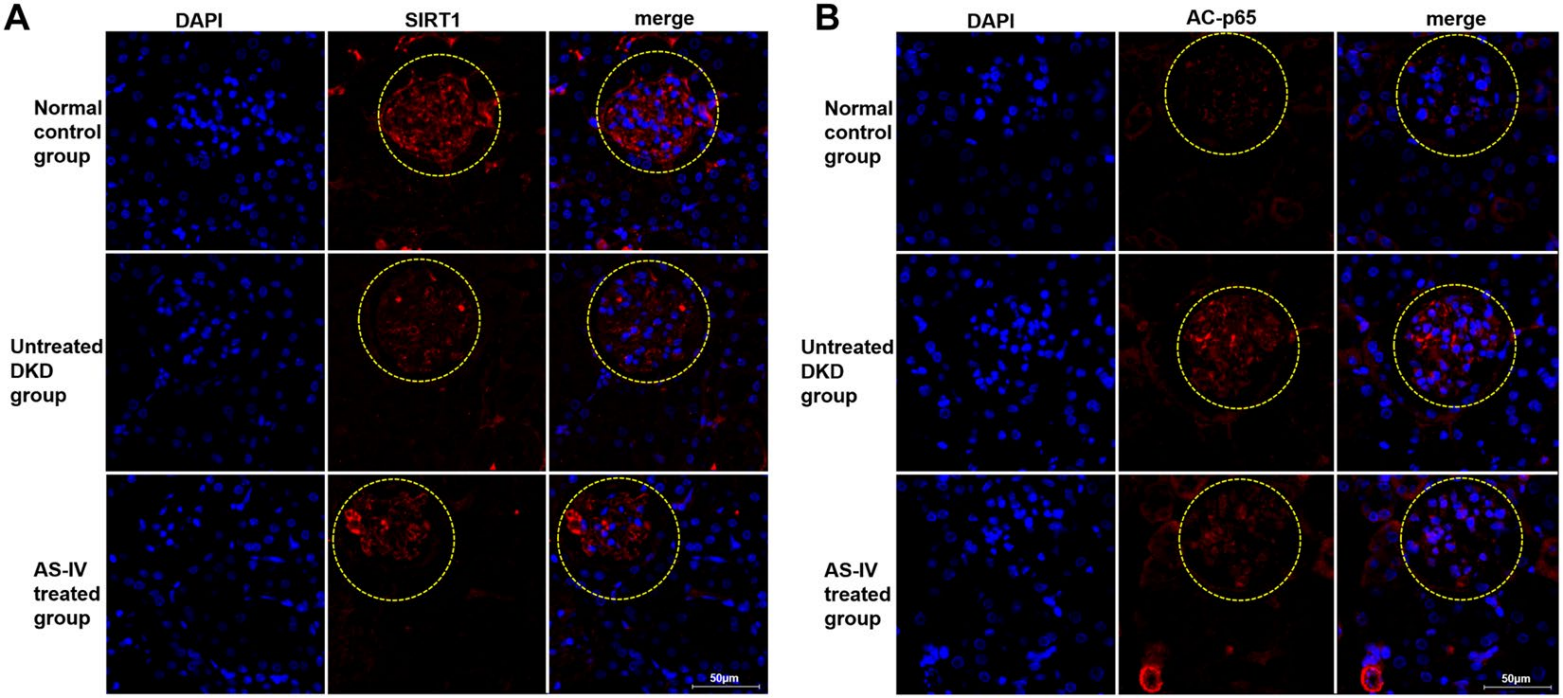
*In vivo* AS-IV effects on SIRT1 and NF-kB p65 expression. (**A**,**B**) The levels of AC-p6 and SIRT1 *in vivo* were analyzed by immunofluorescence assay.

Furthermore, SIRT1 and p65 acetylation expressions were detected *in vivo* by immunofluorescence assay. The findings revealed that AS-IV exposure raised expressions of SIRT1 while decreasing levels of p65 acetylation in contrast to untreated DKD mice (Fig. 4A,B).

### AS-IV effects on the modulation of the SIRT1-NF-κB pathway on autophagy in high glucose-induced podocyte EMT

**Figure 5:**
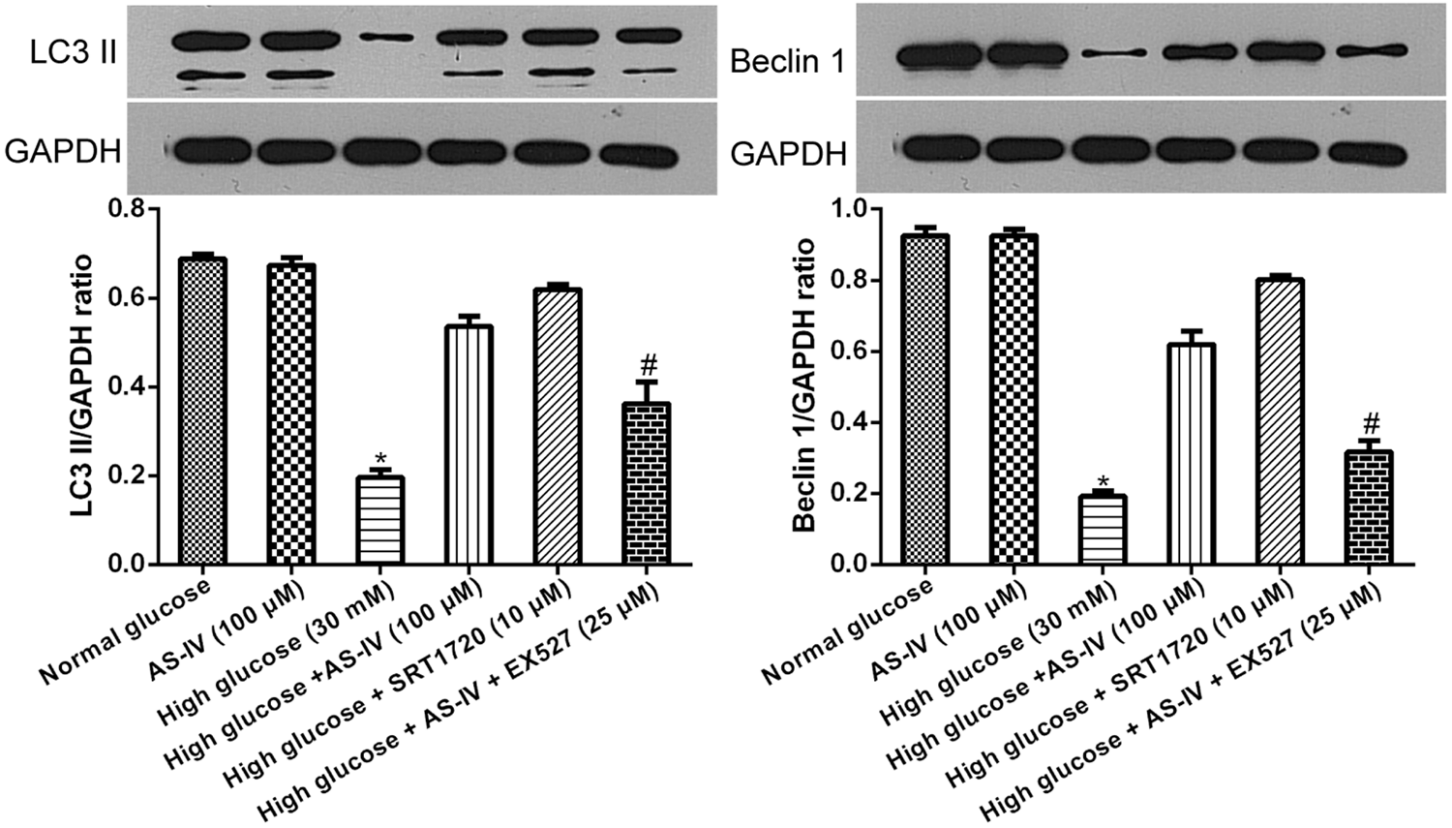
AS-IV effects on SIRT1-induced inactivation of NF-κB signaling results in autophagy in hyperglycaemia-triggered podocyte EMT. Podocytes were pretreated with EX527 or SRT1720 after a one hour exposure to hyperglycaemic conditions, and then incubated with or without of AS-IV (100 μM) for 48 hours. Beclin 1 and LC3II protein levels were quantified via Western blotting. The molecular weight of the proteins: LC3 II, 16 kDa; Beclin 1, 60 kDa. Data is presented as mean ± SD. n = 3. *Compared with normal glucose cohort, or AS-IV cohort, or high glucose plus AS-IV cohort, or high glucose plus SRT1720 cohort, P < 0.05; ^#^compared with high glucose plus AS-IV cohort, P < 0.05.

Previous reports have suggested that SIRT1 deacetylation was involved in NF-κB pathway regulated autophagy^27^. Further examination of NF-κB inactivation in podocyte autophagy induced by SIRT1, podocytes in hyperglycaemic conditions were exposed to SRT1720 or EX527 and subsequently incubated for 48 hours with or without AS-IV. Expressions of the autophagy makers Beclin 1 and LC3 II were then analyzed via Western blotting. High glucose suppressed both Beclin I and LC3 II expressions during podocyte EMT. These changes were restored by AS-IV (Fig. 5). However, the AS-IV effects were abolished upon EX527 treatment but augmented in the presence of SRT1720 (Fig. 5). Our data indicates that AS-IV enhanced autophagy through promotion of SIRT1-facilitated deacetylation of the NF-κB p65 submit.

### AS-IV-induced autophagy effects hyperglycemic-induced podocyte EMT

**Figure 6:**
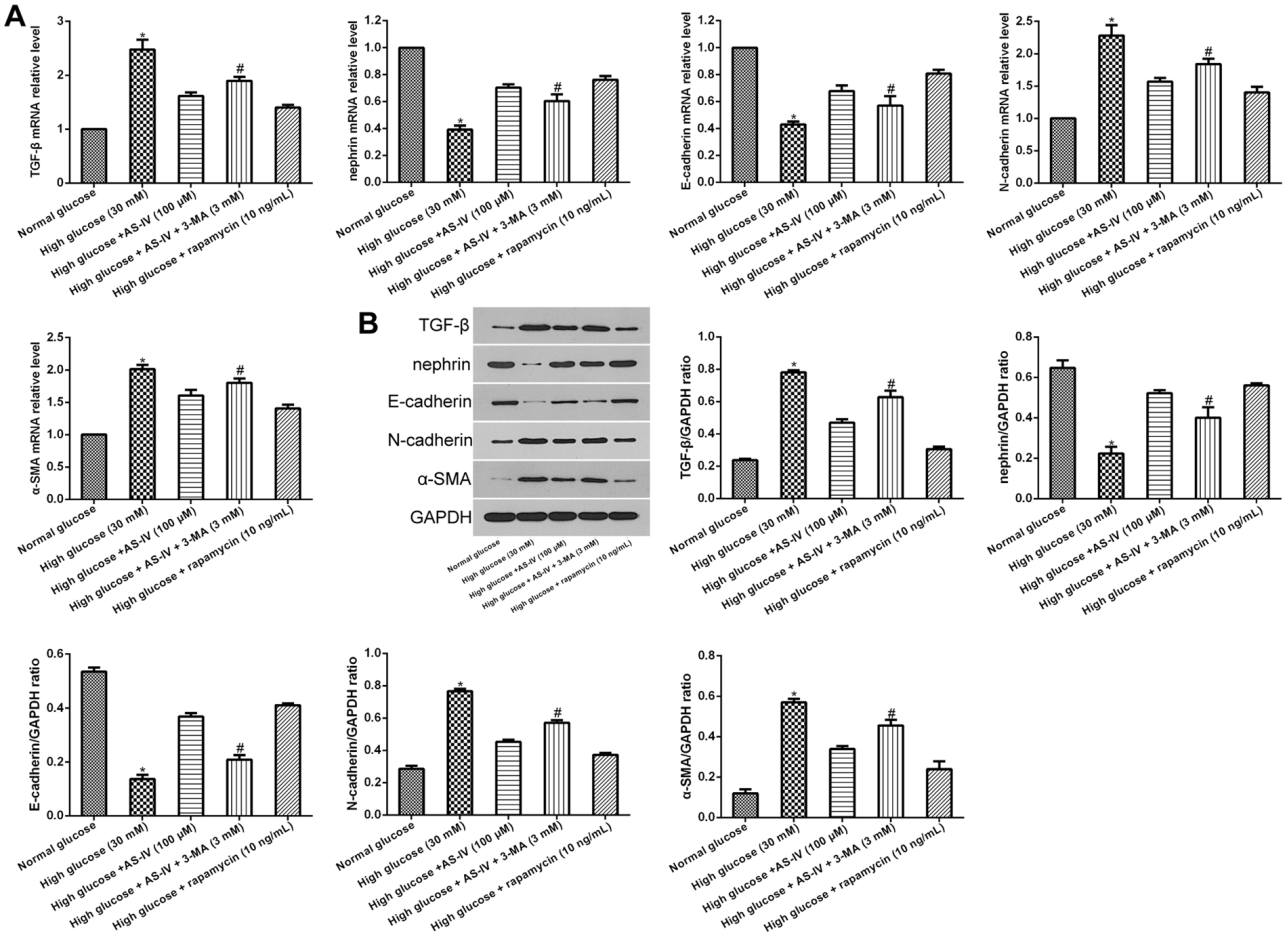
AS-IV-induced autophagy effects on hyperglycemia-triggered podocyte EMT. (**A**,**B**) Podocytes were treated with rapamycin or 3-MA after hyperglycemic exposure, followed by incubation with or without AS-IV (100 μM) for 48 h. (**A**) mRNA levels of TGF-β, nephrin E-cadherin, α-SMA and N-cadherin were quantified using real-time PCR. (**B**) The protein levels of TGF-β, nephrin E-cadherin, N-cadherin and α-SMA were measured by Western blotting. Data is presented as mean ± SD. n = 3. *Compared with normal glucose cohort, or high glucose plus AS-IV cohort, or high glucose plus rapamycin cohort, P < 0.05; ^#^compared with high glucose plus AS-IV cohort, P < 0.05.

Investigation into the impact of AS-IV-triggered autophagy on podocyte EMT was done by first treating podocytes in hyperglycaemic conditions with either inhibitor 3-Methyladenine (3-MA) or with autophagy activator rapamycin, followed by incubation with or without AS-IV for 48 hours. Both rapamycin and AS-IV attenuated expressions of TGF-β, N-cadherin and α-SMA, while increasing E-cadherin and nephrin levels in podocytes cultured in hyperglycaemic conditions. These changes were also reversed in the presence of 3-MA (Fig. 6A,B), suggesting that AS-IV ameliorated high glucose-induced podocyte EMT by enhancing autophagy.

### AS-IV effects on renal function and morphology in diabetic KK-Ay mice

**Figure 7:**
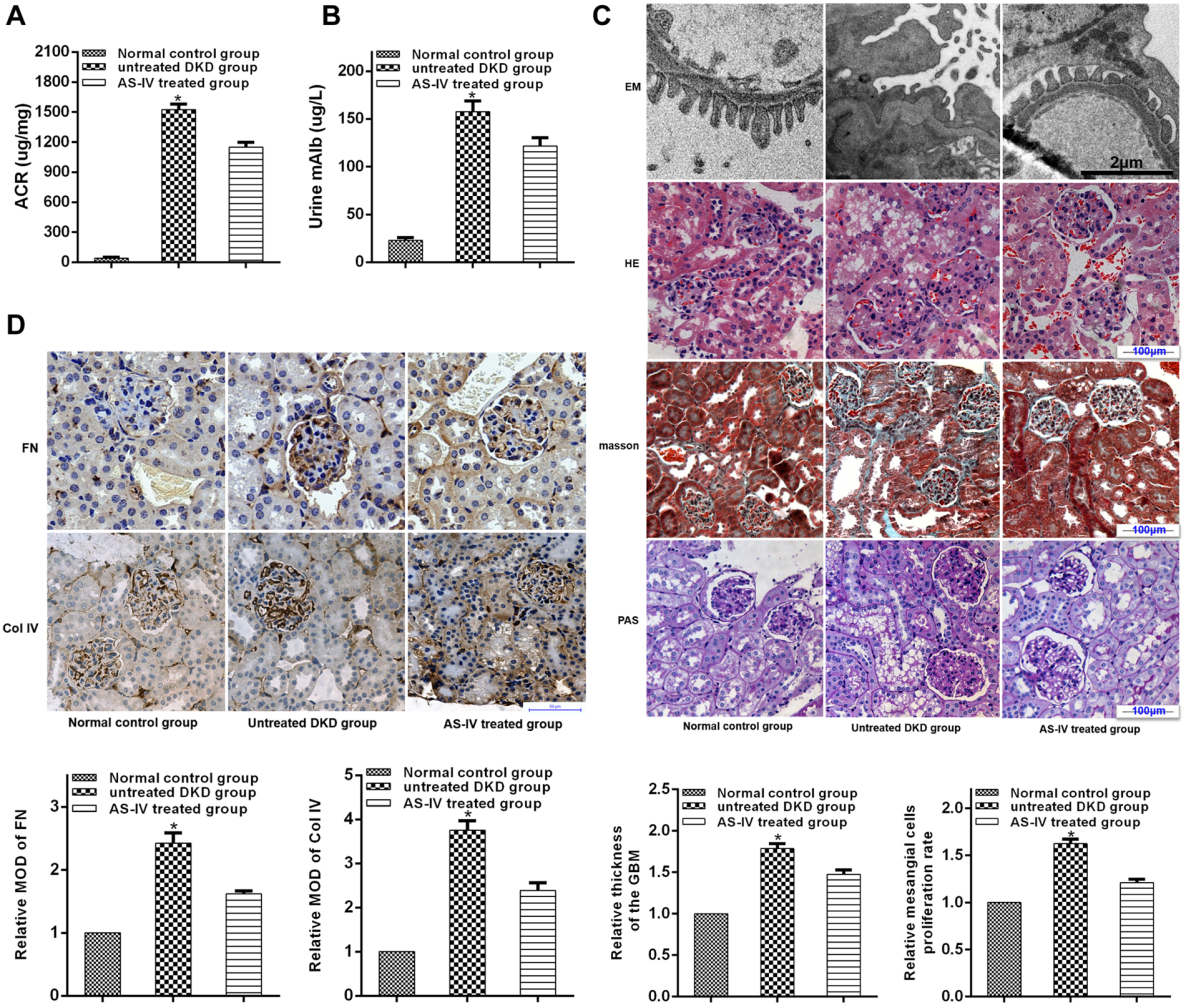
AS-IV effects on renal function and morphology in diabetic KK-Ay mice. (**A**,**B**) Renal function was evaluated by the levels of ACR and mAlb. (**C**) Light and electronic microscopy (EM) was used to observe renal morphologies. Representative photographs are shown for EM, hematoxylin-eosin (HE), masson and Periodic Acid-Schiff (PAS) staining. (**D**) AS-IV effects on Col IV and FN expressions in diabetic KK-Ay mice. Representative photographs of IHC are depicted for FN and col-IV. Data is presented as mean ± SD. n = 12. *Compared with normal control cohort or AS-IV treated cohort, P < 0.05.

Further examination on the impact of AS-IV on renal function and morphology was carried out by evaluation of urine microalbuminuria (mAlb) levels and ACR values as well as histological examination of renal morphology done on both light and electronic microscopy in diabetic KK-Ay mice. AS-IV treated mice showed diminished ACR and lower microalbuminuria in contrast to untreated DKD mice (Fig. 7A,B). In addition, AS-IV treatment augmented fusion of podocytes foot process and improved structural discordances in podocytes, attenuated glomerular basement membrane (GBM) thickening, while reducing over-production of ECM, mesangial proliferation and renal fibrosis in contrast to untreated DKD mice (Fig. 7C). Furthermore, since the overproduction of extracellular matrix (ECM) is a histological marker of renal fibrosis in DKD, an analysis of the expressions of primary ECM components (Col IV and FN) was done to evaluate renal fibrosis. Finding revealed that both FN and Col IV levels were significantly diminished in the kidneys of AS-IV mice in contrast to DKD mice (Fig. 7D). Taken together, AS-IV enhanced renal morphology and function while decreasing severity of renal fibrosis in diabetic KK-Ay mice.

## Discussion

The progression of proteinuria in diabetic kidney disease (DKD) is associated with functional and morphological changes in the podocytes. EMT, a type of podocyte injury, can be triggered by several pathological factors, such as autophagy and oxidative stress. Recent studies have emphasized the role of autophagy in EMT since autophagy can act both as an activator or inhibitor of tumor development in different cancer types^15–17^. While several studies demonstrated the relationship between AS-IV and autophagy in various cells including podocytes^21,28,29^, few have indicated the relationship between autophagy and podocyte EMT. Therefore, we studied the inhibitory effect of autophagy on podocyte EMT, and found that while high glucose concentrations attenuated expressions of autophagy markers LC3-II and Beclin1, AS-IV exposure was able to halt this effect, indicating that autophagy in hyperglycemia-triggered podocyte injury was enhanced by AS-IV.

Accumulating evidence suggest a direct connection between autophagy and EMT^15–17^, and numerous studies have focused on understanding the role of autophagic signaling in regulating EMT. Recent studies have reported that SIRT1 directly regulates autophagy^30,31^, with SIRT1-knockout mice demonstrating suppressed capabilities of podocyte autophagy^30^. SIRT1 can regulate autophagy by de-acetylating several proteins associated with autophagy, such as Atg5 and Atg7^31^. In addition, NF-kB signaling was also found to be a critical influencer of autophagy. Wei *et al*. illustrated that NF-κB signal activation inhibited autophagy in high glucose-stimulated podocyte apoptosis by downregulating LC3-II^13^. Similarly, our results showed that high glucose diminished podocyte autophagy while augmenting NF-κB signaling. These changes were rescued via treatment with AS-IV. We hypothesize that AS-IV inhibited NF-κB signal activation through stimulation of SIRT1-mediated deacetylation. Indeed, podocytes treated with the SIRT1 activator SRT1720 showed increased autophagy and weakened NF-κB p65 acetylation, as with AS-IV treatment, and these effects were abolished by the SIRT1 inhibitor EX527. A previous study suggested that SIRT1 protected against podocyte dysfunction via inhibition of NF-κB p65 subunit acetylation^12^, which is consistent with our results. Taken together, our data showed that AS-IV functions to activate SIRT1, which then inhibits NF-κB p65 subunit acetylation, all of which culminates in enhanced autophagy in hyperglycaemia-induced podocyte injury. AS-IV may stimulate SIRT1 activity by allosteric interaction^32^, an area that would benefit from additional research.

**Figure 8:**
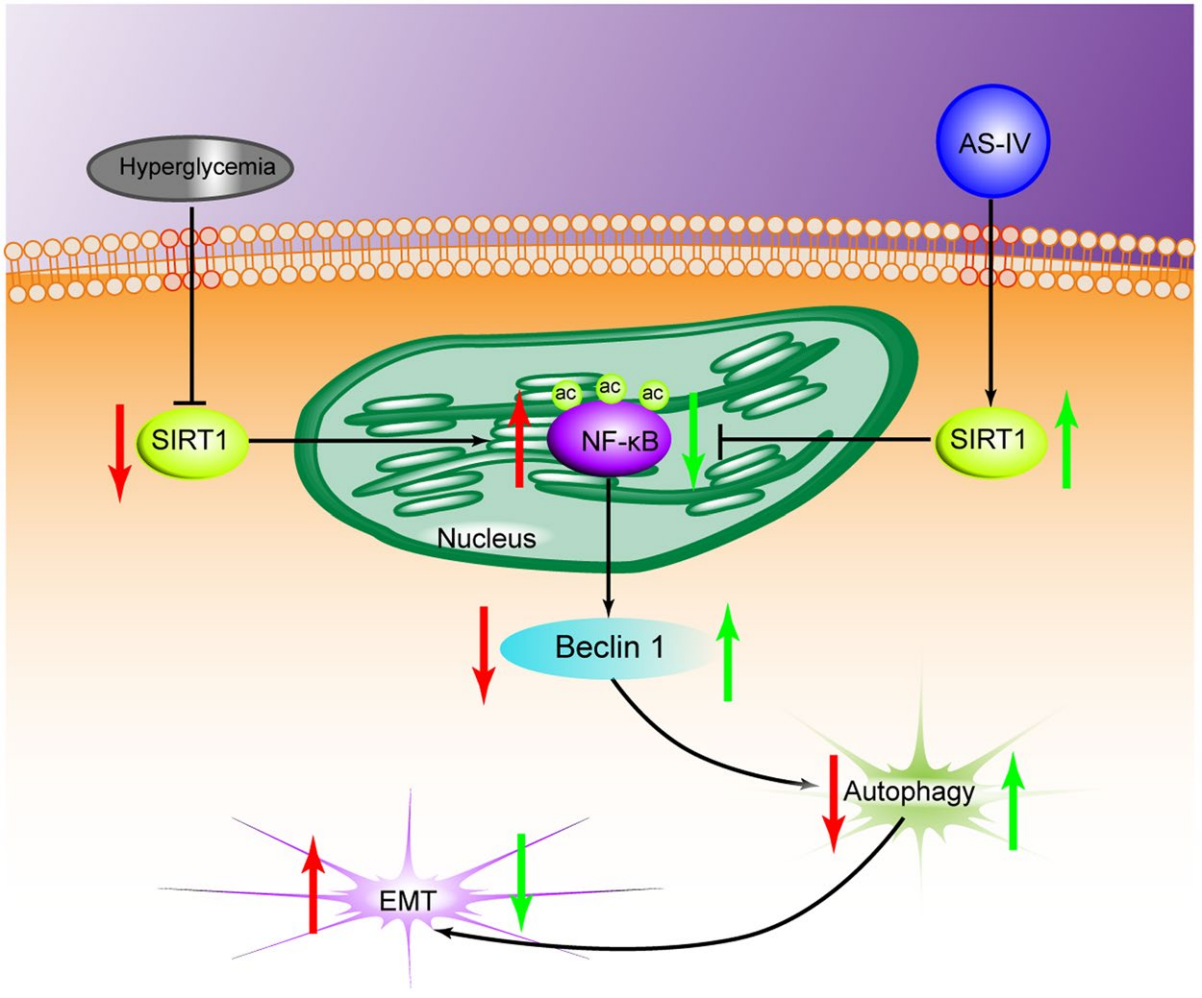
A diagram depicting the proposed roles of AS-IV in hyperglycaemia-triggered podocyte EMT. In response to the harmful stimuli of high glucose, podocytes undergo EMT. During this state, SIRT1 expression is reduced, which further leads to the inhibition of deacetylation activity. The NF-kB subunit p65 enters the nucleus and is acetylated (AC-p65). The increased expression of AC-p65 leads to the decrease of autophagy. AS-IV treatment increases the expression of SIRT1, which leads to the increase of deacetylation activity. AC-p65 is consequently down-regulated, thus increasing levels of autophagy. Therefore, AS-IV can ameliorate podocyte EMT through enhancement of autophagy by SIRT1 deacetylation of the p65 subunit of NF-kB.

A number of reports have suggested that SIRT1 may function as an autophagy inducer of EMT of different cells^33–35^. Sun *et al*. reported that SIRT1 promoted EMT in melanoma cells by enhancing autophagy-associated E-cadherin degradation^33^. Ding *et al*. demonstrated that alisertib inhibited ovarian cancer cells EMT and induced autophagy by increasing SIRT1 levels^34^. A previous study showed that SIRT1 inhibited gastric cancer growth by downregulating NF-κB^35^. Therefore, SIRT1 mediated autophagy may be a potential target for inhibiting EMT during cancer progression. Our indicate that both autophagy activators and AS-IV treatment improved podocyte EMT under hyperglycemic conditions, and that co-treatment with autophagy inhibitor and AS-IV reversed this effect. We hypothesize that AS-IV protected against podocyte injury induced by high glucose through enhancement of autophagy. Furthermore, we found that AS-IV improved podocyte EMT, which was reversed by autophagy inhibitor. In this study, we used a polygenic KK-Ay mice model of human type 2 diabetes mellitus which possessed diabetic nephropathy lesions that were mimics of human DKD^36^. We found that AS-IV treatment preserved normal renal morphology while improving function as well as reducing the degree of renal fibrosis in the diabetic KK-Ay mice. Taken together, these reports suggest that podocyte EMT may be affected positively by AS-IV through augmentation of autophagy via activation of the SIRT1-NF-κB pathway (Fig. 8). In addition, the autophagy inhibitor failed to completely reverse that protective effects of AS-IV on high glucose-induced podocyte EMT. One possible reason could be that the AS-IV effects of on podocyte EMT could be dependent in other signaling pathways, such as the TGF-β1/Smads pathway^37^ and the Akt/GSK-3β/β-catenin pathway^38^, both of which will benefit from further research. In conclusion, our study indicates that AS-IV reverses high glucose-induced podocyte EMT by enhancing autophagy in DKD, and provides a basis for additional studies examining AS-IV as a therapeutic agent for glomerular diseases.

## Materials and Methods

### Reagents

AS-IV (purity by high-performance liquid chromatography [HPLC] ≥ 98%) was obtained from Sigma-ALDRICH (74777, USA). Rapamycin (S1039), EX527 (S1541), SRT1720 (S1129), 3-MA (S2767) and PDTC (S3633) were bought from Selleck Chemicals (USA). Rabbit polyclonal anti-TGF-β1 (ab92486), anti-N-cadherin (ab18203), anti-SIRT1 (ab12193), anti-Beclin 1 (ab62557), anti-LC3 II (ab48394), anti-collagen IV (Col IV) (ab6586), anti-fibronectin (FN) (ab2413), anti-alpha smooth muscle actin (α-SMA) (ab5694), anti-NF-kB p65 (ab16502) and anti-NF-kB p65 (acetyl K310) (Ac-p65) (ab19870) antibodies were procured from Abcam (UK). Rabbit polyclonal anti-nephrin antibody (NBP1-77303) was provided by Novus Biologicals (USA). Rabbit monoclonal anti-E-cadherin antibody (3195 S) was ordered from Cell Signaling Technology (USA).

### Cell culture

Immortalized mouse podocyte cell line was provided by BeNa Culture Collection (China). Podocytes were grown in the RPMI 1640 medium supplemented with 10% recombinant interferon gamma (IFN-γ) and 10% fetal bovine serum under atmospheric conditions of 5% CO2 at 33 °C. Cells were cultured at 37 °C for more than 7 days in order to induce cellular differentiation and until 80% confluence was achieved. Cells were then synchronized in serum-free conditions for 24 hours, after which the cells were used for the respective experiments.

Nine groups of podocytes were designated: Normal-concentration glucose cohort (5.6 mmol/L glucose + 24.5 mmol/L mannitol); High-concentration glucose cohort (30 mmol/L glucose); AS-IV (100 µmol/L) group; high glucose + AS-IV cohort (the concentration of AS-IV = 25, 50, or 100 µmol/L); high glucose + SRT1720 (10 µmol/L) cohort; high glucose + AS-IV (100 µmol/L) + EX527 (25 µmol/L) cohort; high glucose + PDTC (10 µmol/L) cohort; high glucose + rapamycin (10 ng/mL) cohort; high glucose + AS-IV (100 µmol/L) + 3-MA (3 mmol/L) cohort.

### Animals and treatment

Polygenic KK-Ay mice models were selected as they possessed renal lesions that closely resemble those found in DKD of human type 2 diabetes mellitus^39^. These mice models were used to evaluate AS-IV effects on renal function and fibrosis in DKD. 8-week-old male KK-Ay mice and male C57BL/6 J mice were purchased from Beijing Huafukang Bioscience Co. Inc (China). All procedures conformed to the Guide for the Care and Use of Laboratory Animals by the National Institute of Health and was approved by the Institutional Animal Care and Use Committee at Capital Medical University. Mice were reared with ad libitum access to water, consistent humidity (70%) and room temperature (24 °C) and were exposed to a fixed light to dark cycle. KK-Ay mice were fed with high-fat diet (HFD; 58% fat, 16.4% protein and 25.6% carbohydrates) for 4 weeks to induce DKD as previously described^40^ while C57BL/6 J mice were fed a standard diet (12% fat, 28% protein and 60% carbohydrate). KK-Ay mice were selected for further experimentation their urine albumin creatinine ratio (ACR) was ≥300 ug/mg and their random blood glucose was ≥16.7 mM. The DKD KK-Ay mice were then randomly divided into the control/DKD group (n = 12, gavaged with aqua distillate) and treatment/AS-IV group (n = 12, gavaged with AS-IV at 40 mg/kg/day)^41,42^. C57BL/6 J mice functioned as the normal control (NC) group (n = 12, gavaged with aqua distillate). 12 weeks later, 24-hour urine was harvested. The mice were killed, and renal tissues were collected for further studies.

### Immunohistochemistry and -fluorescence staining

4% paraformaldehyde was used to fixate renal tissues which were then paraffin-embedded and sliced into sections for immunohistochemistry (IHC) and Immunofluorescence (IF) analysis. In brief, slices were deparaffinized, dehydrated subjected antigen retrieval. After blocking the endogenous peroxidase activity with 3% hydrogen peroxide, the sections were blocked with 5% goat serum for 30 minutes, and then incubated overnight with primary antibodies at 4 °C. This was followed by secondary antibody incubation for 1 hour at 37 °C. Lastly, sections were counterstained with DAPI for the nuclei. Images of stained cells were captured using a fluorescence microscope. For IHC, the primary antibodies used were the rabbit anti-Col-IV antibody (1:100) and rabbit anti-FN antibody (1:100). For IF, the primary antibodies used were the rabbit anti-nephrin antibody (1:100), rabbit anti- Ac-p65 (1:200), rabbit anti-α-SIRT1 (1:200) and rabbit anti-α-SMA (1:100).

### Real-time PCR analysis

Total RNA extraction from cells and kidney tissues was carried out using the TRIzol reagent (Invitrogen, USA) in compliance to manufacturer’s instructions. Detection of podocyte nuclear p65 expressions was done using a nuclear fractionation kit (Solarbio, SN0020, China) to extract nuclear RNA. Real-time PCR primers were designed as reported by past literature^39^. Relative expressions were assessed with the comparative cycle threshold (Ct) method (2^−ΔΔCT^)^43^. Relative expressions of p65, SIRT1, α-SMA and nephrin were normalized to GAPDH expression. All mRNA expression analyses were done with the results of a minimum of three independent experiments.

### Western blotting

Total cellular and kidney protein were extracted, subjected to SDS-PAGE, and electroblotte onto PVDF membranes. The latter was blocked with 5% nonfat dry milk and underwent an overnight incubation with primary antibody at 4 °C followed by horseradish peroxidase-conjugated secondary antibody (1:1,000, Beyotime, China). Rabbit polyclonal primary antibodies and dilutions are as follows: TGF-β (1:1000), nephrin (1:5000), E-cadherin (1:1000), N-cadherin (1:1000), α-SMA (1:1000), Ac-p65(1:400), SIRT1(1:1000), Beclin 1 (1:1000) and LC3 II(1:3000). Subsequently, enhanced chemiluminescence was used for band detection.

### SIRT1 deacetylase activity assay

Nuclear protein of podocytes was extracted by using a CelLytic™ NuCLEAR™ Extraction Kit (Sigma-Aldrich, NXTRACT, USA) as recommended by the manufacturer. Then the deacetylase activity of SIRT1 in nuclear protein of podocytes was detected with a SIRT1 activity assay (Abcam, ab156065, UK) based on instructions of the manufacturer. Measurements of fluorescence intensity was carried out at 340 nm excitation and 460 nm emission using a microtiter plate fluorometer.

### Statistical methods

SPSS software (IBM, USA) enabled statistical analysis. All data is presented as ±SD of at least three independent experiments. Student’s unpaired t test allowed comparison of two data groups and multiple group comparison was done using one-way ANOVA. P-values of less than 0.05 were taken to indicate statistical significance.
